# Analysis of mRNA Expression and DNA Methylation Datasets According to the Genomic Distribution of CpG Sites in Osteoarthritis

**DOI:** 10.3389/fgene.2021.618803

**Published:** 2021-04-15

**Authors:** Peng Yi, Xiongfeng Xu, Jiawei Yao, Bo Qiu

**Affiliations:** Department of Orthopedic Surgery, Renmin Hospital of Wuhan University, Wuhan, China

**Keywords:** DNA methylation, gene transcription, genomic regions, chondrocytes, osteoarthritis

## Abstract

Abstract Objectives Transcriptional changes in cartilage can impact function by causing degradation such as that which occurs during the development of osteoarthritis (OA). Epigenetic regulation may be a key factor leading to transcriptional changes in OA. In this study, we performed a combined analysis of DNA methylation and gene expression microarray datasets and identified key transcription factors (TFs) central to the regulation of gene expression in OA. Methods A DNA methylation profile dataset (GSE63106) and a gene expression profiling dataset (GSE114007) were extracted from the Gene Expression Omnibus (GEO). We used ChAMP methylation analysis and the Limma package to identify differentially methylation genes (DMGs) and differentially expressed genes (DEGs) from normal and human knee cartilage samples in OA. Function enrichment analysis of DMGs was conducted using the DAVID database. A combined analysis of DEGs and DMGs was conducted to identify key TFs in OA. We then validated the mRNA expression of selected TFs in normal and OA cartilage by RT-qPCR. Primary chondrocytes were cultured and treated with the DNA methylation inhibitor 5-Aza-2-deoxycytidine (5-Aza) for functional validation. Results We identified 2,170 differential methylation sites (DMS) containing 1005 genes and 1986 DEGs between normal human and OA cartilage. Functional analysis of DMGs revealed that focal adhesion, extracellular matrix (ECM)-receptor interactions, the PI3K-Akt signaling pathway, and the FoxO signaling pathway were involved in OA. Integrated analysis showed a subset of 17 TFs. Four TFs (ELF3, SOX11, RARA, and FOXD2) were validated. RT-qPCR results showed the mRNA expression of SOX11, RARA, and FOXD2 were consistent with the results from the mRNA expression data. However, the expression of ELF3 could not be validated. Upon 5-Aza-2′-deoxycytidine (5-Aza) treatment, the mRNA levels of ELF3 and SOX11 were down-regulated, whilst RARA was up-regulated, and FOXD2 showed no significant change in expression level. Conclusions the effect of DNA methylation on the transcriptional regulation is related to the distribution of methylated sites across the genome. Epigenetic studies on the positions of DMS in transcriptional units can inform a better understanding of the function of DNA methylation and its transcription regulation.

## Introduction

Osteoarthritis (OA) is a common degenerative disease characterized by the destruction of articular cartilage and subchondral bone (Fathollahi et al., 2019). OA is a multifactorial disease with several associated risk factors including sex, age, trauma, obesity, joint injury, joint overuse, and genetic factors such as altered transcriptional control and epigenetic regulation. In OA, transcriptional changes in cartilage can alter its function and cause degradation (Bortoluzzi et al., 2018).

Recent studies have demonstrated that epigenetic regulation may be a key factor leading to transcriptional changes in OA chondrocytes (Barter and Young, 2013; Im and Choi, 2013). DNA methylation is a form of chemical modification in DNA that covalently binds methyl group on the cytosine 5′ carbon of cytosine-phosphate-guanine (CpG) dinucleotides in the genome. Previous studies have found many differentially or aberrantly methylated sites in OA that cause common pathogenesis including degradation of the extracellular matrix (ECM), collagen breakdown, and activation of the inflammatory response (Steinberg et al., 2018). However, the exact mechanisms by which methylation impacts gene transcription and ultimately leads to pathological changes in OA remain to be fully determined.

Previously, it has been reported that the analysis of transcription factors (TFs) is crucial toward understanding biological processes mediated by the epigenetic regulation (Zhu et al., 2016). Recently, methods for genome-wide methylation studies have been developed and applied to demonstrate that the distribution of methylation sites across the genome can regulate gene expression (Harris et al., 2010). Amongst these sites, methylation in gene promoters acts to inhibit transcription. This process has been verified in a range of genes involved in the pathogenesis of OA including genes associated with the ECM(*COL9A1*), matrix-degradation (*MMP13*), inflammation (*IL-1*β), and oxidative stress (*SOD2*) (Hashimoto et al., 2009; Scott et al., 2010; Bui et al., 2012; Imagawa et al., 2014). These genes showed methylation changes in promoter CpG sites in OA chondrocytes and were negatively correlated at the transcriptional level. It is known that epigenetic changes are essential for tissue development, differentiation, and cell viability (Jones, 2012). However, the functions of methylation sites in enhancers, insulators, and gene body regions, and the detailed mechanisms that affect gene transcription remain to be fully determined.

In this study, we analyzed microarray data (Illumina Infinium Human Methylation 450) from normal and OA human knee cartilage to determine the distribution of CpG sites across the genome. These data were combined with mRNA expression analysis to determine differentially expressed transcription factors (DETFs). Cartilage samples were collected from patients with OA and used to validate changes in mRNA expression levels of the identified TFs. We then performed *in vitro* experiments to verify the DNA methylation patterns that regulate gene expression.

## Materials and Methods

### Human Cartilage Samples and Primary Chondrocytes Cell Ccultures

Knee articular cartilage was obtained from three patients who were undergoing knee replacement surgery due to primary knee OA in July 2020. Ethical approval was obtained from the Committee of the Renmin Hospital of Wuhan University and all samples were obtained under informed consent from each patient. All standard biosecurity and institutional safety procedures have been adhered to in all the experiment procedures in this article. Previous studies established a three-region disease progression model system for OA of the knee. The model includes the outer lateral tibial plateau (oLT), the inner lateral tibial plateau (iLT), and the inner medial tibial plateau (iMT) regions of the knee that represent the early, intermediate and late stages of OA (Zhang et al., 2016). Therefore, we obtained normal cartilage and OA cartilage from the oLT and the iMT regions.

Human primary chondrocyte cells (cat. no.CP-H096; Pcocell Life Science and Technology Co., Ltd.) were cultured in DMEM/F12 medium (cat. no. SH30023.01; Hyclone) supplemented with 10% fetal bovine serum (FBS), chondrocyte growth additive, vitamin supplements and 1% double-antibody (penicillin/streptomycin Solution). Cells were incubated at 37°C in 5% CO_2_. We started the experiment when the chondrocytes were in the logarithmic growth phase. Primary chondrocytes were incubated with or without 10 μM 5-Aza-2′-deoxycytidine (5-Aza) (cat. no. A119533; Aladdin) for 4 days (Iliopoulos et al., 2007; Zhao et al., 2017). Total RNA was extracted from the two groups of cultured chondrocytes to determine the mRNA levels of selected TFs.

### DNA Methylation Profiling and Gene Expression Data

The NCBI Gene Expression Omnibus (GEO) database was searched to obtain a DNA methylation profiling dataset (GSE63106) and a gene expression profiling dataset (GSE114007). The inclusion criteria for the selection of samples were; (1) Samples derived from the OA or healthy control cartilage tissue, (2) Sample size was >20, and (3) Samples where the data had been analyzed to assess functional TFs (or transcriptionally functional CpG). GSE63106 was used to collect data from the preserved and affected cartilage samples obtained from 17 knees and 14 hip joints undergoing joint replacement surgery due to primary OA. GSE114007 was used to collect data from 20 OA and 18 normal human knee cartilage tissues.

### Analysis of DNA Methylation Profiling Data

We used the ChAMP methylation analysis package in R (v3.6.0) to analyze GSE63106 in all subsequent processes (Morris et al., 2014). Firstly, we loaded intensity data into R (v3.6.0) after filtering unnecessary CpG using the champQC function and generated several plots using the QC.GUI function for data visualization. We then used the champ.norm function with BMIQ to perform a type-II probe normalization of the raw data (Teschendorff et al., 2013). From these analyses, we obtained the adjusted *P*-values as outputs, log fold change (FC), and the genomic regions between the normal and OA groups. Differentially methylation genes (DMGs) were those including CpG sites with abs (logFC) >0.1 and adjusted *P*-values < 0.05. The percentage of differential methylation sites (DMS) and the array sites (after filtering) in different genomic regions were statistically analyzed.

### Gene Ontology and KEGG Pathway Enrichment Analysis

Gene ontology (GO) and the KEGG signaling pathway enrichment analysis was conducted on DMGs using the DAVID bioinformatics database. Enriched GO terms and KEGG signaling pathways with *P*-values < 0.05 were deemed as significant.

### Identification of Differentially Expressed Genes

As the gene expression profiling datasets extracted from GSE114007 are normalized expression matrices, we did not repeat quantile normalization. To ensure normalization, we used the boxplot function in R to check data normalization. The raw data were analyzed using the R BIOCONDUCTOR packages. Differentially expressed genes (DEGs) were identified through the LIMMA package with the cut-off criteria set as abs (logFC) >1 and adjusted *P*-value < 0.05.

### Identification of Functional Transcription Factors

A list of human TFs and their motifs were downloaded from the “HumanTFs” Website (Lambert et al., 2018). We then compared the DEGs and DMGs with the list of human TFs to identify DETFs whose encoding genes showed simultaneous differential methylation patterns.

### Reverse Transcription Quantitative PCR (RT–qPCR)

Total RNA was extracted using Trizol Reagent (cat. no. G3013; Tiangen Biotech, Co., Ltd.) according to the manufacturer’s protocol and transcribed into cDNA using a RevertAid First Strand cDNA Synthesis Kit (cat. no. #K1622, Thermo). The thermocycling conditions were as follows: initial denaturation at 95°C for 30 sec, followed by 40 cycles of 95°C for 15 s, 60°C for 60°s. The melting curve was generated (dissociation, 60°C 95°C at 15 s) to determine normality. FastStart Universal SYBR Green Master (Rox) (Roche) was used for the quantitative analysis. GADPH was used as the internal reference gene. The 2^–Δ^ ^Δ^ ^Cq^ method was used to analyze the results. The primer sequences of the genes are presented in Table 1.

**TABLE 1:** Primer sequences of the genes used for RT-qPCR.

	Primer sequence (5′-3′)	
**Target Gene**	**Forward**	**Reverse**
*GAPDH*	CAATGACCCCTTCATTGACC	GACAAGCTTCCCGTTCTCAG
*ELF3*	GATCTCCGAGCAAGAGCGTAG	GTCTTCGACCAGAACTGGGG
*FOXD2*	GCGCCAAAGCCTTCTACG	GAGCGATAGAGCCCGCTTAG
*RARA*	CACACACCTGAGCAGCATCAC	CGGTCCTTTGGTCAAGCAGT
*SOX11*	AGCGGAGGAGGTTTTCAGTG	TTCCATTCGGTCTCGCCAAA

### Statistical Analysis

All data were expressed as the means ± SEM. Data between the two groups were compared using an unpaired Student’s *t*-test. Graphs were generated using GraphPad Prism 7 software and *P*-values of <0.05 considered statistically significant.

## Results

### Identification of DMGs in Preserved and Affected Cartilage From 17 Knees Joint Samples

**FIGURE 1:**
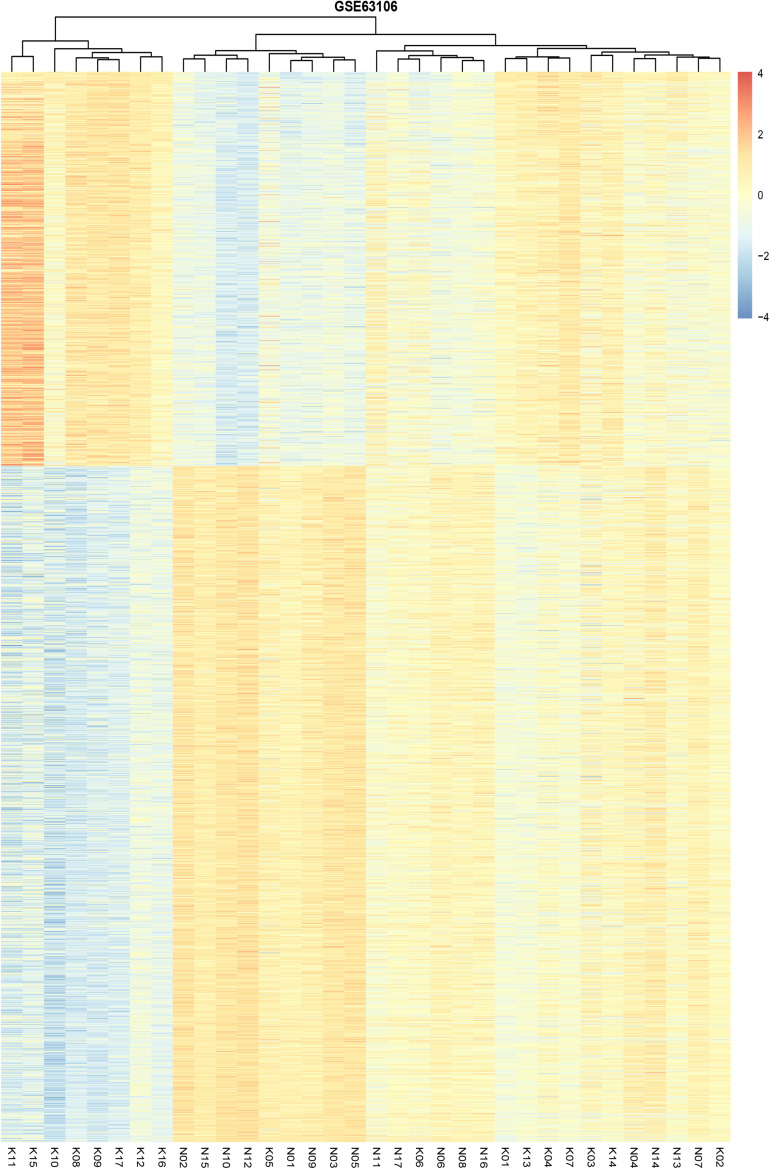
Unsupervised hierarchical clustering of the methylation levels of the DMS from the preserved and affected cartilage, based on the cut-off criteria of abs(logFC) >0.1 and adjusted *P*-value < 0.05.

We identified 2,170 DMS containing 1005 genes between preserved and affected cartilage. A complete list of the DMS is shown in Supplementary Table 1. Amongst these DMS, 799 sites were hypomethylated and 1,371 site hypermethylated in the OA cartilage. Unsupervised clustering of the 2,170 DMS revealed that samples from normal and OA cartilage could be divided into two main clusters (Figure 1). The first cluster contained eight knee OA samples. The second cluster contained nine knee OA samples and 17 normal samples. Interestingly, the knee OA samples revealed two separate clusters. A previous study reporting the DNA methylome in OA of the knee and hip revealed that OA samples segregated into two groups (Rushton et al., 2014). These results suggest that OA may be divided into two distinct subtypes based on differences in their DNA methylation. The observed differential clustering in the same joint may provide insight toward the development of future diagnostic and therapeutic approaches in OA. However, a critical and challenging question in cluster analysis is whether the identified clusters represent important underlying structure or are artifacts of natural sampling variation (Kimes et al., 2017).

### Function Enrichment Analysis of DMGs

**FIGURE 2:**
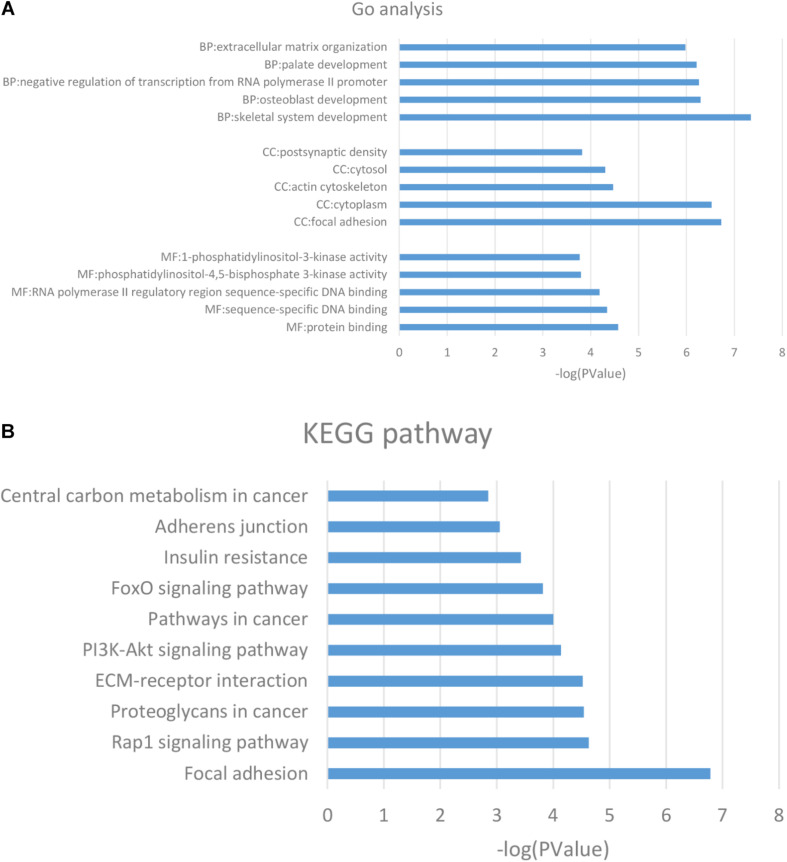
**(A)** GO analysis of the DMGs. **(B)** KEGG pathway analysis of the DMGs. The columns are represented as the negative log10 of the *P*-value. DMGs: differentially methylated genes; KEGG: Kyoto Encyclopedia of Genes and Genomes.

GO analysis showed the common DMGs were mainly enriched for biological processes including the skeletal system development, osteoblast development, negative regulation of transcription from RNA polymerase II promoter, and ECM organization. For molecular functions, the common DMGs were enriched in phosphatidylinositol-4,5-bisphosphate 3-kinase and 1-phosphatidylinositol-3-kinase activity. For cellular components, the common DMGs were mainly enriched in focal adhesion, the cytoplasm and the actin cytoskeleton (Figure 2A). A complete list of GO analysis is presented in Supplementary Table 2. KEGG pathway analysis demonstrated that common DMGs were mainly enriched in focal adhesion and ECM-receptor interactions, and the PI3K-Akt and FoxO signaling pathways (Figure 2B). A complete list of KEGG pathway analysis is shown in Supplementary Table 3.

### Identification and Characterization of DEGs

**FIGURE 3:**
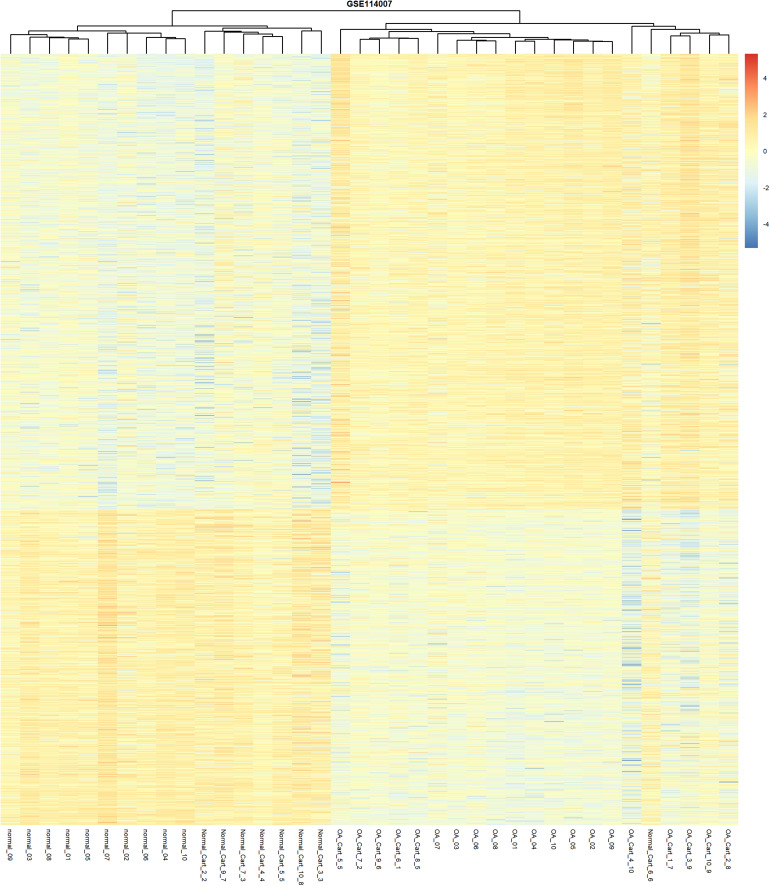
Unsupervised hierarchical clustering of the DEGs, based on their expression levels, from the normal and OA articular cartilage samples using the cut-off criteria of abs (logFC) >1 and adjusted *P*-value < 0.05.

A total of 1986 DEGs were identified between the OA and the normal human cartilage. A full list of DEGs is shown in Supplementary Table 4. Amongst these DEGs, 1174 were upregulated and 812 were downregulated in OA. Unsupervised clustering of the 1986 DEGs revealed that samples from normal and OA cartilage could be divided into two main clusters (Figure 3). The first cluster contained 17 normal samples. The second cluster contained 20 knee OA samples and 1 normal sample. Interestingly, the knee OA samples were divided into two different clusters based on their mRNA expression just like the hierarchical clustering of DNA methylation. The specific significance of the differential clustering observed in the same joint deserves further discussion.

### Statistical Analysis of CpG Sites

Statistical analysis of the number and rate of DMS and the array sites (after screening) was performed in different gene sub-regions. The seven different sub-regions included Transcriptional initiation sites (TSS)200, TSS1500, 1stExon, 3′UTR, 5′UTR, intergenic region (IGR), and gene body. As shown in Table 2, it was observed that 38% of the DMS were enriched in the IGR. In the intragenic regions, 41% of the DMS were significantly enriched in the gene body as opposed to only 17% included in the promoter regions (including TSS200, TSS1500, 5′UTR, and the 1stExon of genes).

**TABLE 2:** DMS and Array sites (after filtering) in different genomic regions.

Genomic regions	DMS	DMS rate	Array sites (after filtering)	Array Rate (after filtering)
3’UTR	83	4%	15005	4%
5’UTR	179	8%	37756	9%
1stExon	23	1%	19832	5%
Gene Body	886	41%	139677	34%
TSS200	52	2%	46675	11%
TSS1500	132	6%	60212	14%
IGR	815	38%	94216	23%
island	125	6%	132924	32%
opensea	1420	65%	144283	35%
shelf	242	11%	38337	9%
shore	383	18%	97829	24%
Enhancer	1189	55%	94497	23%
No enhancer	981	45%	318876	77%

*UTR-Untranslated Region; TSS200: region from TSS to-200 bp upstream of TSS; TSS1500:200-1,500 bp upstream from TSS; Shore: regions 0–2 kb from CpG islands; shelf: regions 2–4 kb from CpG islands; open sea: CpG sites in the genome that do not have a specific designation.*

In addition, we separately analyzed the proportion of DMS and array sites enriched in enhancers. Our results showed that 55% of the DMS were enriched in the enhancer, whilst only 23% were enriched in the array sites. We also analyzed their positions relative to CpG islands (CGI). The positions relative to a CGI included the shore, shelf, and open sea regions. DMS on CGI accounted for 6% whilst the percentage for the array sites was 32%. These data were consistent with previous studies including data from the Human Epigenome Project that showed 80% of the CpG dinucleotides in the human genome are methylated, whereas CGI are usually unmethylated (Brena et al., 2006).

### Integrative Analysis of DNA Methylation and Gene Expression

The DEGs and DMGs were compared against the list of human TFs. From the DNA expression profiling dataset, we identified 168 DETFs in 1986 DEGs. Of these 168 DETFs, 21 encoding genes exhibited differential methylation that harbored at least one DMS. Four of these DETFs (*HMG2A*, *ALX4*, *RORA*, and *TBX3*) were excluded from the study as their encoding genes corresponded with several CpG sites, of which some were hypermethylated and others hypomethylated. The remaining seventeen TFs are shown in Table 3 that included nine hypermethylated genes of which two were up-regulated and seven were down-regulated, and eight hypomethylated genes of which three were up-regulated and five were down-regulated.

**TABLE 3:** DETFs harboring DMS in knee articular cartilage from normal and OA patients.

Methylation Status in OA	TFs	Gene expression status in OA	Genomic regions	logFC of GSE63106	logFC of GSE114007
Hyper-methylated	*SOX11*	Upregulated	3’UTR	0.114052191	3.830859342
	*ATOH8*	Downregulated	Body	0.156007596	−2.082568147
	*MSX2*	Upregulated	TSS1500	0.134644281	1.303540248
	*PITX1*	Downregulated	3’UTR, Body	0.154268857	−1.638461524
	*RARA*	Downregulated	1stExon	0.106699436	−2.309531101
	*PRDM16*	Downregulated	Body	0.162060676	−1.769999863
	*ELF3*	Downregulated	TSS200	0.111024436	−3.022557431
	*FOXD2*	Downregulated	1stExon,3’UTR	0.109470333	−1.184733905
	*TBX4*	Downregulated	TSS200,TSS1500, Body	0.15388695	−1.554387492
Hypo-methylated	*LEF1*	Upregulated	Body	−0.100907626	2.020807828
	*PPARG*	Upregulated	Body	−0.129022244	1.599195067
	*RFX8*	Upregulated	TSS1500	−0.174751917	3.439724382
	*TBX5*	Downregulated	Body	−0.205976918	−1.661135603
	*ARID5B*	Downregulated	Body	−0.105378211	−1.201323891
	*MLXIP*	Downregulated	Body	−0.119453723	−1.459126795
	*NFIL3*	Downregulated	5’UTR	−0.193317129	−2.892963016
	*BCL6*	Downregulated	5’UTR	−0.131035527	−1.530121946

### Changes in the Gene Transcription Profiles of Primary Chondrocytes Following Treatment With DNA Methylation Inhibitors

**FIGURE 4:**
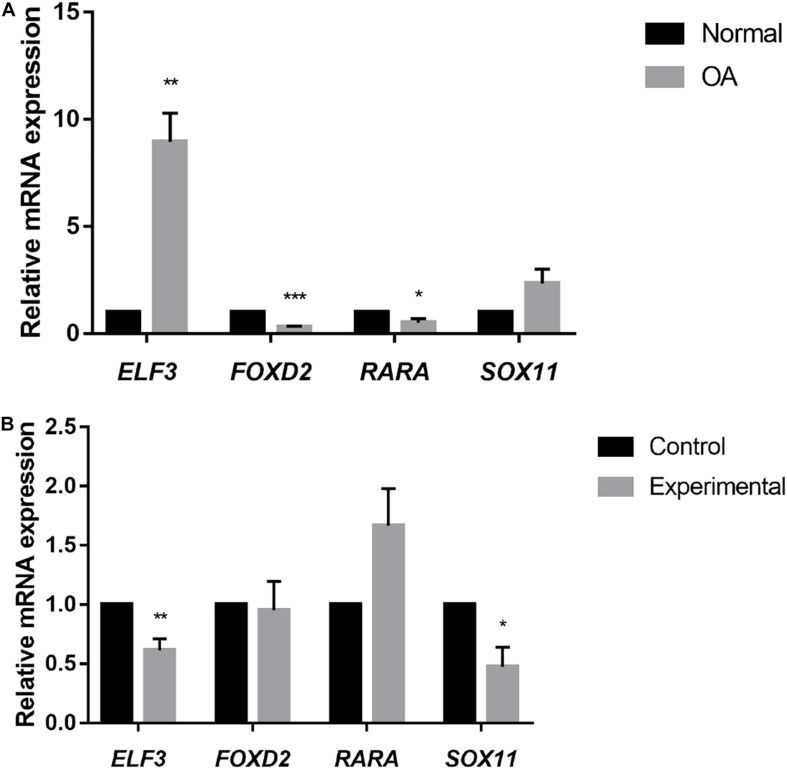
**(A)** Relative mRNA expression of selected TFs in normal and OA knee articular cartilage, assessed by RT-qPCR. *P < 0.05, **P < 0.01, ***P < 0.001. **(B)** Relative mRNA expression of selected TFs in cultured primary human chondrocytes (*N* = 3) treated with 5-Aza (10 μM) or vehicle for 4 days. Values are expressed as mean ± SEM. *P < 0.05, **P < 0.01, ***P < 0.001.

Previous genome-wide methylation studies have shown that promoter methylation negatively correlates with gene expression, whilst methylation in the gene body and 3′UTR is positively correlated with gene expression (Mishra and Guda, 2017). To further explore how the methylation of different genomic regions affects gene transcription, we chose DETFs that conformed to these changes for further investigation including *SOX11*, *RARA*,*ELF3*, and *FOXD2*. The mRNA expression of selected TFs was then validated. *ELF3* and *SOX11* were up-regulated in the cartilage of OA patients, whilst *RARA* and *FOXD2* were down-regulated (Figure 4A). Exception for *ELF3*, the mRNA expression of *SOX11*, *RARA*, and *FOXD2* were consistent with the results analyzed from the mRNA expression data. Upon treatment with 5-Aza, *ELF3*, and *SOX11* mRNA levels decreased, whilst *RARA* mRNA levels increased and *FOXD2* showed no significant change (Figure 4B). These data indicated that DNA demethylation can affect the mRNA expression levels of these particular TFs.

## Discussion

Recently, epigenetic regulation has been shown to play an important role in the pathogenesis of OA (den Hollander et al., 2014). In particular, DNA methylation can have a profound impact on the development of OA. In a study by Roach et al. (2005) the abnormal expression of ECM-degrading enzymes including *MMP*-*3*, *MMP-9*, *MMP-13*, and *ADAMTS-4* in OA chondrocytes were shown to be associated with loss of DNA methylation in their promoter region of these genes. However, the mechanisms driving these epigenetic regulatory processes and the observed phenotypes are yet to be determined.

Genome-wide expression profiling analysis has shown that abnormal transcription is essential for the development of OA (Aigner et al., 2006; Karlsson et al., 2010). Previous studies identified key TFs in the pathogenesis of OA such as *SOX9*, by analyzing DNA methylation in combination with mRNA expression. *SOX9* is expressed by mesenchymal stem cells and can promote the expression of matrix proteins in cartilage (Zhang et al., 2019). A previous study showed an increase in *SOX9* expression that in OA cartilage was affected by its promoter methylation status. 5-Aza could then reverse the decreased levels of *SOX9* gene expression (Kim et al., 2013).

*SOX9* can also interact with methylated DNA. Imagawa et al. demonstrated that the DNA methylation status of *COL9A1* could influence the binding of *SOX9* to DNA. This restrained *SOX9*-driven promoter activation and down-regulated the expression of *COL9A1* (Imagawa et al., 2014). Also, recent progress in epigenetic research has highlighted TFs as a new class of readers and effectors of DNA methylation (Zhu et al., 2016). The analysis of TFs is therefore crucial to explaining the underlying biological processes mediated by epigenetic regulation.

We performed a statistical analysis of CpG sites enriched in different gene sub-regions showing that most DMS were enriched in the IGR (38%) and gene body (41%). Only 17% of DMS were included in the promoter regions. The distance between enhancers and promoters is highly variable. Enhancers are mostly CpG-poor and play an important role in controlling gene expression during development and cell functioning (Lister et al., 2009). The proportion of DMS in enhancer regions (55%) was significantly higher than that in array sites (23% after screening), indicating that epigenetic modifications were more likely to occur in CpG-poor genomic regions (Thurman et al., 2012; Ziller et al., 2013). These data also suggested that the analysis of specific genomic regions during the development of OA may contribute to the understanding of epigenetic functions and their influence on the pathogenesis of OA.

Genome-wide studies have provided detailed information about methylation patterns showing that CpG sites outside TSS have a more complex effect on gene transcription than previously thought. Previous studies focused on the methylation levels of gene promoters. However, gene body methylation remains to be fully characterized. Most studies have focused on CGI at TSS and so DNA methylation is often described as an epigenetic marker for silencing gene expression (Jones, 2012).

In the study of DNA methylation of a single gene in OA, the promoter of *COL10A1* was hypomethylation during chondrocyte hypertrophy, and maturation and was associated with increased *COL10A1* expression (Zimmermann et al., 2008). Similarly, the methylation levels of CpG sites in the promoters of many metalloproteinases including *MMP2*, *MMP9*, *MMP13*, and *ADAMTS4*, were reduced and led to up-regulated gene expression in OA cartilage (Roach et al., 2005; Cheung et al., 2009). Methylation in CpG-poor regions of the genome may be more worthy of study than promoter regions.

From the early stages of DNA methylation, it has been known that gene body methylation is characteristic of transcriptional genes (Wolf et al., 1984). Gene bodies are mostly CpG-poor, widely methylated and contain multiple repeating and translocation elements (Jones, 2012). Recently, it was confirmed that there is a widely positive correlation between gene methylation and transcription on the X chromosome (Hellman and Chess, 2007). However, as early as 1999, researchers paradoxically proposed that promoter methylation is inversely correlated with expression, whilst gene body methylation is positively correlated with expression (Jones, 1999).

Gene body methylation is thought to be associated with the regulation of gene splicing. Exons have higher levels of methylation compared to introns, and methylation is more likely to change at exon-intron boundaries (Laurent et al., 2010). However, recent genome-wide studies of methylation have clearly shown that methylation in promoters and 5′UTR are mostly negatively correlated with gene expression, whilst methylation in genes and 3′UTR are positively correlated (Mishra and Guda, 2017).

In this study, we performed a combined analysis of DNA methylation and gene expression microarray datasets. We identified 17 TFs that exhibited simultaneously different methylation and expression patterns between normal and OA cartilage samples. We then validated the mRNA expression of selected TFs and performed functional validation in primary chondrocytes treated with 5-Aza.

Analysis of the mRNA expression data showed that *ELF3* was down-regulated in OA cartilage. However, RT-qPCR results showed that *ELF3* was up-regulated in OA cartilage. These data suggest that the results obtained from the database analysis are not entirely accurate and require further verification.

We treated cultured human primary chondrocytes with 5-Aza that resulted in the down-regulation of *ELF3* mRNA. Although this result was contrary to our expectations, it indicated that methylation in promoters does not always result in gene silencing. Also, previous studies have linked *ELF3* dysregulation to epigenetic changes. *ELF3* is a member of the ELF subset of the ETS family of TFs. A previous study showed that increased *ELF3* levels in OA inhibited *COL2A1* by directly binding to ETS sites in the *COL2A1* promoter and then binding to the HMG domain of *SOX9*. This resulted in the repression of CBP/p300 driven HAT activity. The dysregulation of *ELF3* was associated with hypermethylation of the *ELF3* proximal gene promoter (Otero et al., 2017) which is further supported by our study.

Another study revealed that *ELF3* could also activate *IL-1*β and *TNF*α-mediated *MMP13* transcription by binding to the *MMP13* proximal promoter induced by *IL-1*β (Otero et al., 2012). These studies provide strong evidence that *ELF3* is a valuable TF in the pathogenesis of OA. Also, these data revealed that DNA methylation status can not only change the transcription of TFs, but also alter the binding of TFs to DNA target sequences that regulate the expression of genes and cause phenotypic changes.

*SOX11* belongs to the group C of the Sox family that is composed of many supergenes with conserved motifs of the HMG box (Kavyanifar et al., 2018). Decreased levels of *SOX6* and *SOX9* are associated with the phenotypic change of chondrocytes in OA (Sock et al., 2004). Recent studies have also shown that *SOX11* is up-regulated in OA tissue and can promote OA by induction of *TNF-*α (Xu et al., 2019). Our results suggest that the increased level of *SOX11* mRNA in OA tissue may be related to methylation in the 3′UTR region of the gene. In addition, the DMS of *RARA* was located in the 1stExon region, while the DMS of *FOXD2* was found in both 1stExon and 3′UTR. It may explain the increased mRNA expression level of *RARA* after 5-Aza treatment, while the *FOXD2* showed no significant change. *RARA* and *FOXD2* do not have an obvious role in OA pathogenesis highlighting this as an important area for further studies.

Our findings on *ELF3* indicate that hypermethylation of gene promoters may increase gene transcription. Although methylation in promoters is usually manifested as gene silencing, it is not clear whether methylation is the cause or the result of gene silencing. Studies have found that inactivation of the X chromosome results in methylation of the *Hprt* gene (Lock et al., 1987). The link between DNA methylation and transcription is likely to be significantly more complex than initially realized. Although DNA methylation cannot always explain changes in transcription, a better understanding of methylation changes in OA cartilage tissue may facilitate the development of novel therapeutic approaches by preventing or reversing epigenetic changes.

There were limitations to our study. Firstly, we did not investigate the normal cartilage from the oLT regions. Undoubtedly, our results would be more representative if we could add qPCR or western-blot to validate the oLT samples. Nevertheless, previous studies of the pathology and transcriptome showed that the oLT regions are very similar to normal. Thus, the oLT regions could serve as a suitable alternative to normal control, which could also reduce the inter-individual variations (Liu et al., 2018). Therefore, the measurement of the cartilage from the oLT regions is considered non-essential. Secondly, the sample size of cartilage tissue we acquired were small. In the future, we will pay more attention to the sample size.

For the first time, we analyzed DNA methylation and mRNA expression data in OA patients according to the distribution of CpG sites across the genome. Our comprehensive analysis identified a subset of DETFs whose gene transcriptional changes may be related to DNA methylation status. These TFs are potential biomarkers and drug targets, and merit further study in the pathogenesis of OA. Also, we found that the distribution of DMS in the genome had obvious characteristics. Moreover, the effect of DNA methylation on transcription is related to the distribution of CpG sites. Further studies on the interaction between epigenetic modification and transcription will provide more effective insights into the pathogenesis of OA.

## Data Availability Statement

Publicly available datasets were analyzed in this study. This data can be found here: https://www.ncbi.nlm.nih.gov/geo/query/acc.cgi?acc=GSE63106; https://www.ncbi.nlm.nih.gov/geo/query/acc.cgi?acc=GSE114007&lt.

## Ethics Statement

The studies involving human participants were reviewed and approved by the Committee of the Renmin Hospital of Wuhan University. The patients/participants provided their written informed consent to participate in this study.

## Author Contributions

PY and BQ designed the study. PY, XX, and JY acquired and interpreted the data. PY analyzed the data and was a major contributor in writing the manuscript. XX and JY prepared figures and tables. BQ prepared the manuscript and supervised the study. All authors read and approved the final manuscript.

## Conflict of Interest

The authors declare that the research was conducted in the absence of any commercial or financial relationships that could be construed as a potential conflict of interest.
